# Medical Management of Cyclosporine-Induced Gingival Overgrowth Using Oral Azithromycin in Six Dogs

**DOI:** 10.3390/vetsci2010013

**Published:** 2015-02-05

**Authors:** Alison Diesel, Karen Moriello

**Affiliations:** 1 Department of Small Animal Clinical Sciences, College of Veterinary Medicine and Biomedical Sciences, Texas A&M University, College Station, TX 77843, USA; 2 Department of Medical Sciences, School of Veterinary Medicine, 2015 Linden Drive West, University of Wisconsin, Madison, WI 53706, USA; E-Mail: moriellk@svm.vetmed.wisc.edu

**Keywords:** cyclosporine, gingival overgrowth, azithromycin, dog

## Abstract

Gingival overgrowth is an uncommon adverse effect of cyclosporine administration in veterinary species. In people, gingival overgrowth is a common complication of cyclosporine administration for immunosuppression, generally following transplant procedures. Azithromycin has been used successfully for managing gingival overgrowth in human transplant patients when cyclosporine administration cannot be reduced or discontinued. This case series describes six dogs being administered cyclosporine for various dermatologic diseases that developed gingival overgrowth. The dogs were prescribed systemic azithromycin, with or without concurrent dose reduction of cyclosporine. Oral administration of 6.6–10.8 mg/kg of azithromycin once daily for 4–14 weeks was effective for complete clinical resolution of gingival overgrowth. In most cases, gingival overgrowth did not recur even with continued cyclosporine administration long-term. Adverse events of long-term azithromycin administration did not occur in any of the dogs. This series highlights a potentially beneficial medical treatment option for gingival overgrowth even when cyclosporine dose reduction is not possible or elected, without the need for surgical resection of proliferative gingival tissue.

## 1. Introduction

Cyclosporine is an immunosuppressive medication used to treat a variety of dermatological diseases in veterinary medication. This calcinurin inhibitor works by binding cyclophilin in the cytoplasm of primarily T cells which thereby inhibits activation. This results in decreased production of various inflammatory cytokines including interleukin (IL)-2, 3, 4 and tumor necrosis factor alpha. The drug was originally designed for use in human transplant patients and has been used since the 1970s [1]. Human formulations have been used in veterinary medicine since the 1980s [2], however it wasn’t until 2003 that the first veterinary approved product (Atopica^®^, Novartis Animal Health) was available. The modified form is absorbed more effectively and predictably in small animals leading to enhanced bioavailability [3]. Although the most common adverse effects reported in veterinary patients are gastrointestinal issues (vomiting, diarrhea, nausea, anorexia), numerous other complications with cyclosporine therapy have been noted [3].

Gingival overgrowth is a well-documented side effect of cyclosporine administration in both dogs and people [3,4,5,6,7,8]. The exact incidence of occurrence is unknown, but is thought to affect approximately 2%–3% of canine patients treated for atopic dermatitis with the medication [3,9]. At higher dose levels, incidence has been reported as high as 75% [6]. Duration of therapy, genetics, and breed factors have also been purported to play a role in development of cyclosporine-induced gingival overgrowth. Other than cosmetic concerns, gingival overgrowth can result in the formation of dental pseudopockets resulting in plaque accumulation and potential tooth detachment [10]. Additionally, pain and difficulty chewing along with gingival bleeding have also been noted in canine patients [6]. Historically, cyclosporine dose reduction and/or surgical resection of excess gingival tissue have been considered to be the best option for management of gingival overgrowth in canine patients.

In people, gingival overgrowth is a common complication of cyclosporine administration for immunosuppression, generally following transplant procedures. Azithromycin has been used successfully in managing gingival overgrowth in humans when cyclosporine administration cannot be reduced or discontinued [11,12]. This macrolide (azalid family) antibiotic is thought to reduce gingival overgrowth by means of antimicrobial effects, reduction of inflammation, and possible disruption of protein synthesis by fibroblasts [13]. A single study exists in the veterinary literature evaluating the use of azithromycin (both as an oral capsule and toothpaste form) for management of cyclosporine-induced gingival overgrowth in dogs [14]. In this prospective study, only a single parameter (gingival sulcus depth) was improved with a four week administration of azithromycin, however a single dog did experience complete resolution of gingival overgrowth during the study time period (eight weeks).

The purpose of this retrospective case series is to review the records of dogs that had been treated with oral azithromycin (Zithromax^®^; Pfizer, New York, NY, USA) for cyclosporine-induced gingival overgrowth prior to the publication of the more recent prospective study. All cases of cyclosporine-induced gingival overgrowth seen by the authors over a five year time frame were included in the review. As much of the evidence regarding this option was previously anecdotal, additional cases were used to further evaluate the potential benefit of this alternative medical therapy over surgical correction, particularly in cases where cyclosporine administration was continued long-term for disease management.

## 2. Case Series

### 2.1. Case #1

**Figure 1:**
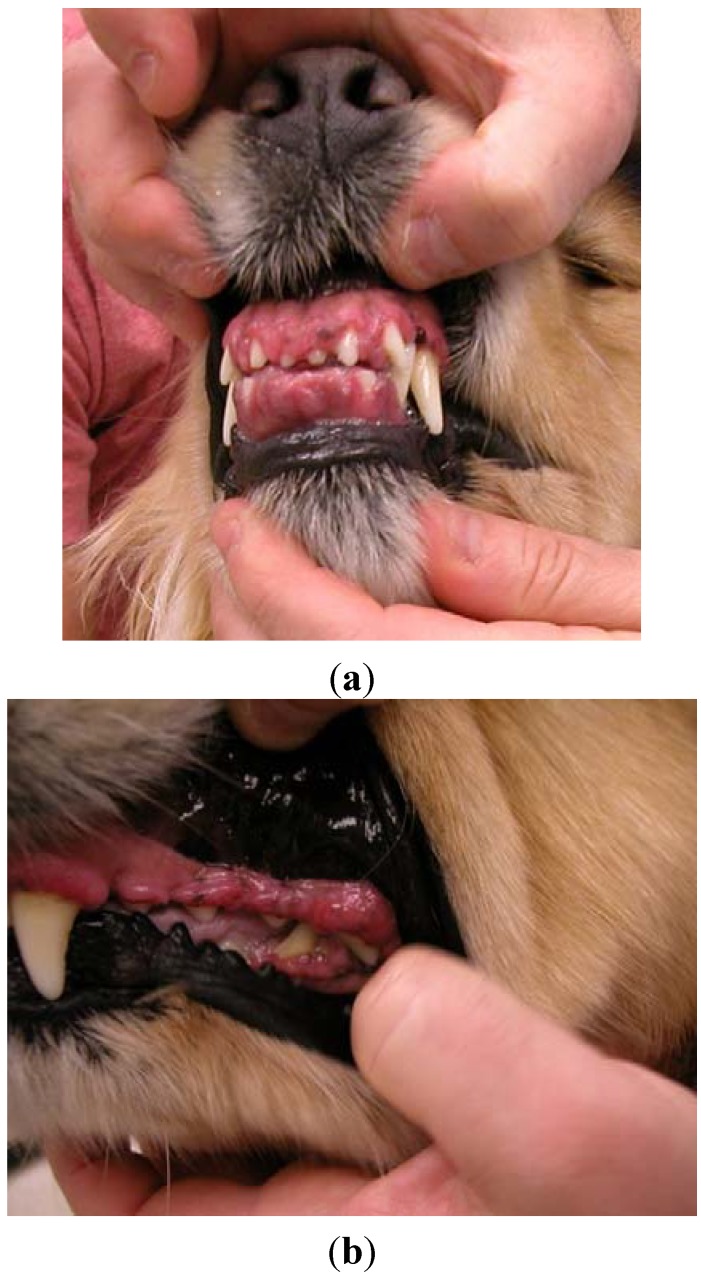
Case #1—Severe gingival overgrowth over the upper and lower incisors (**a**); and premolars and molars (**b**) prior to initiating azithromycin therapy.

A four-year, 26.4 kg female spayed golden retriever dog was presented for atopic dermatitis. Her primary care veterinarian had initiated therapy with modified cyclosporine (human generic) 3.8 mg/kg once daily and concurrent ketoconazole 3.8 mg/kg once daily approximately nine months prior to presentation on referral. The dog presented after owners noted severe halitosis, excessive drooling, “disappearance of her teeth”, and gingival bleeding. The dog’s appetite was not affected. Oral examination revealed marked gingival overgrowth with gingivitis affecting the entire oral cavity. Gingival tissue had proliferated to cover incisors, premolars and molars over all dental arcades (Figure 1a,b).

Intradermal allergy testing was pursued and allergen-specific immunotherapy (ASIT; injectable) was formulated; however due to marked improvement in the dog’s pruritus, the owner requested to continue cyclosporine administration pending ASIT benefits. Both cyclosporine and ketoconazole were changed to alternate day administration. In addition, azithromycin was initiated at 9.6 mg/kg orally once daily.

**Figure 2:**
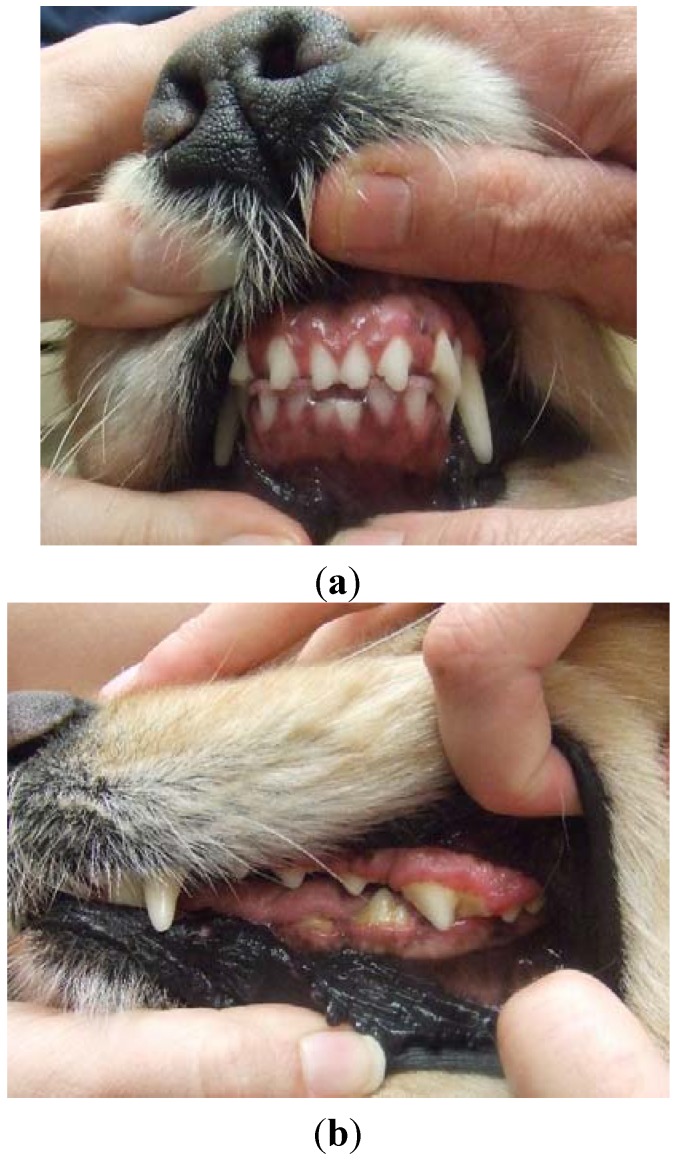
Same dog as in Figure 1(a,b), showing complete clinical remission of gingival overgrowth following 14 weeks of continued azithromycin administration.

At week 4, there was marked improvement in gingival overgrowth as evidenced by less gingival hyperemia and increased visibility of teeth on all arcades (photographed to document visual changes at each visit); however persistent evidence of excessive gingival tissue over the molars was seen. All treatments were continued unchanged. At week 10, gingival overgrowth was still improving. Lower incisors were visible and only mild excessive gingival tissue over upper molars was noted. Azithromycin was continued for an additional 4 weeks at which time full clinical resolution of gingival overgrowth was noted; the dog received a total of 14 weeks of azithromycin therapy. The dog was followed for over six years; gingival overgrowth has not recurred with continued cyclosporine administration (no further dose adjustments) (Figure 2a,b).

### 2.2. Case #2

A 2.5 year, 13.6 kg male castrated Pembroke Welsh corgi dog presented for a follow up examination of atopic dermatitis which had not responded to ASIT after one year of administration. The dog responded favorably to prednisone 0.2–0.4 mg/kg every other day; however the owner was concerned with long-term side effects of continued corticosteroid administration. Modified cyclosporine (human generic) therapy was initiated at a dose of 5.5 mg/kg once daily.

At a nine month follow up examination, moderate gingival overgrowth was noted over all dental arcades, particularly the molars. The owners had not noted this adverse effect however, as the dog did not show any evidence of oral discomfort. Cyclosporine was reduced to 3.7 mg/kg once daily and azithromycin was initiated at 9.3 mg/kg orally once daily. After 12 weeks of azithromycin administration, complete clinical resolution of gingival overgrowth was noted. Cyclosporine administration was continued at the current dose.

Approximately one year later, the dog presented for an annual examination. Mild-moderate gingival overgrowth was again noted over all arcades; excessive gingival tissue extended to almost completely cover the molars and premolars. Azithromycin therapy was repeated; the dog however had lost weight (2 kg) over the last year resulting in an oral azithromycin dose of 10.8 mg/kg once daily. The cyclosporine dose remained the same. Complete clinical resolution of gingival overgrowth was noted after 8 weeks. Recurrence was not noted with continued cyclosporine administration (dose unchanged) over a course of over four years.

### 2.3. Case #3

A three year, 40 kg male castrated German shepherd dog was presented for presumed atopic dermatitis. At the time, corticosteroid administration could not be withdrawn to pursue intradermal allergy testing due to the severity of the dog’s pruritus. Modified cyclosporine (human generic) 5 mg/kg once daily was prescribed. After six weeks of administration, the owners were able to reduce the dose to 2.5 mg/kg once daily to achieve the same clinical effect. Due to marked improvement in the dog’s comfort, the owners elected not to pursue additional allergy testing or allergen specific immunotherapy.

At one year follow-up examination, the dog was reported to be comfortable while receiving cyclosporine 2.5 mg/kg daily. The previous month however, the dog’s primary care veterinarian had noted moderate gingival overgrowth (biopsy confirmed gingival hyperplasia) when the dog was presented for evaluation of oral bleeding when chewing on toys. On examination, there was moderate gingival overgrowth over all dental arcades with pronounced excessive tissue over premolars and molars. Azithromycin 6.6 mg/kg once daily was prescribed with continued cyclosporine administration (dose unchanged). Moderate improvement in gingival overgrowth was noted by week 4 and complete clinical resolution by week 6. Recurrence was not noted one year later with continued cyclosporine administration.

### 2.4. Case #4

An eight year, 28 kg castrated male Labrador retriever dog was presented for a follow up examination of atopic dermatitis. The dog had failed to respond to ASIT, and the pruritus was being managed with modified cyclosporine (Atopica^®^, Novartis Animal Health) 5.3 mg/kg on alternate days for greater than two years. On examination, moderate gingival overgrowth was present along the upper molars and premolars. Azithromycin was prescribed 8.9 mg/kg orally once daily. Complete clinical resolution of gingival overgrowth was appreciated after 6 weeks. Even with continued cyclosporine administration (dose unchanged), recurrence was not seen for over three years.

### 2.5. Case #5

A nine year, 14.1 kg male castrated Boston terrier dog was presented for a follow up examination of atopic dermatitis. After failing to respond to ASIT, the dog had been managed with cyclosporine (Atopica^®^, Novartis Animal Health) administration. Initial dosing of 5 mg/kg once daily had been reduced to 1.8 mg/kg once daily for one year. During that time however, the dog developed severe gingival overgrowth (biopsy confirmed gingival hyperplasia) and the primary care veterinarian performed surgical resection. Moderate gingival overgrowth remained after surgery, impairing the dog’s ability to eat comfortably. When the dog presented for re-evaluation of allergy management on referral, azithromycin was prescribed 8.9 mg/kg once daily; complete clinical resolution was noted by week 7. Recurrence was not noted even with continued cyclosporine administration (dose unchanged) for over a year. Eventually however the owner discontinued cyclosporine administration after initiation of sublingual immunotherapy successfully managed the dog’s pruritus from atopic dermatitis.

### 2.6. Case #6

A nine year, 32 kg male castrated German shepherd dog was presented for evaluation of a perianal fistula. On examination, a single 1cm diameter draining tract was found which extended into the perianal tissue. No communication with the rectum or anal glands was noted. Modified cyclosporine (human generic) 4.6 mg/kg twice daily and ketoconazole 6.3 mg/kg once daily were administered concurrently; dramatic improvement in the clinical appearance was noted within 1–2 weeks of initiation of therapy.

At week 6, there was marked clinical improvement of perianal fistula, however the dog had developed a focal firm swelling of gingival tissue at the rostral maxilla. Biopsy confirmed gingival hyperplasia. Due to the clinical improvement in perianal fistula, the cyclosporine dose was reduced to 4.6 mg/kg once daily with continued concurrent ketoconazole administration. Azithromycin was prescribed 7.8 mg/kg orally once daily. After 4 weeks of therapy, gingival overgrowth showed complete clinical resolution. Dose reduction was continued for cyclosporine until the perianal fistula completely healed, however the lesion eventually returned. Re-initiation of cyclosporine therapy 4.6 mg/kg daily again resulted in resolution of perianal fistula without recurrence of gingival overgrowth for over one year.

## 3. Results and Discussion

The signalment and case specific details are summarized in Table 1. This case series describes canine patients successfully treated for cyclosporine-induced gingival overgrowth with systemic azithromycin administration without the need for surgical resection; the duration of azithromycin therapy was variable ranging from 4 to 14 weeks. In most cases, gingival overgrowth occurred at cyclosporine doses below what is typically used for induction of management of atopic dermatitis (e.g., 5 mg/kg/day), confirming the fact that gingival overgrowth can occur even at low doses in some dogs. All dogs experienced complete clinical remission long-term, documented with prolonged follow-up. Although valid concern exists regarding the use of antibiotics for anti-inflammatory purposes as a contributing factor to the development of bacterial antibiotic resistance, risk is relatively low due to maintained remission long-term without the need for continued antimicrobial therapy.

**Table 1:** Signalment and summary of case specific details.

Case Number	Age, Sex, Breed	Underlying Disease	Duration of CsA Therapy at Diagnosis (Months)	Dose of CsA at Diagnosis (mg/kg)	Changes in CsA Dose Post Diagnosis	Concurrent Drugs During Treatment	Azithromycin Dose	Weeks to Clinical Remission	Follow-Up	Relapse
1	FS, 4 year, golden retriever	AD	9	3.8 q24h	Changed interval to q48 h	Ketoconazole 3.8 mg/kg/day	9.6 mg/kg/day	14 weeks	6 year	No
2	MC, 2.5 year, Pembroke Welsh corgi	AD, failed ASIT	9	5.5 q24h; 4.3 q24h (at time of GO recurrence)	Decrease to 3.7 mg/kg/day; None	None	9.3 mg/kg/day; 10.8 mg/kg/day	12 weeks; 8weeks	4 year	Yes at 1 year, remission induced within 8 weeks of retreatment
3	MC, 3 year, German shepherd	AD	12	2.5 q24h	None	None	6.6 mg/kg/day	6 weeks	1 year	No
4	MC, 8 year Labrador retriever	AD, failed ASIT	24	5.3 q48h	None	None	8.9 mg/kg/day	6 weeks	3 year	No
5	MC, 9 year Boston terrier	AD, failed ASIT	12	1.8 q24h	None	None	8.9 mg/kg/day	7 weeks	1 year; CsA eventually discontinued due to favorable response to SLIT	No
6	MC, 9 year, German shepherd	Perianal Fistula	1.5	4.6 q12h	Decrease CsA to once a day	Ketoconazole 6.3 mg/kg/day	7.8 mg/kg/day	4 weeks	1 year	No

Abbreviations: AD = atopic dermatitis; ASIT = allergen-specific immunotherapy (injectable); CsA = cyclosporine; GO = gingival overgrowth; SLIT = sublingual immunotherapy.

Gingival overgrowth is a reported rare adverse effect of cyclosporine administration in veterinary species. Several reports have noted this adverse finding; however gingival overgrowth was generally reported at dosages much higher than those used for dermatologic conditions (*i.e.*, anywhere from 15–30 mg/kg) [3]. Onset and occurrence of gingival overgrowth may be associated with elevated cyclosporine levels in the blood; trough levels maintained above 400–700 ng/mL for at least 32 weeks resulted in marked gingival overgrowth in a canine renal transplant model [6]. In most cases, cyclosporine levels are not measured when treating veterinary dermatological diseases; rather, clinical improvement is used as therapeutic monitoring. None of the reported cases in this series had cyclosporine drug concentrations evaluated. It would be interesting however to determine whether patients that develop gingival overgrowth secondary to cyclosporine administration also have documented elevations in serum cyclosporine concentrations, even when administered low doses of the medication.

In people, gingival overgrowth is a well-documented complication of organ transplant patients being administered cyclosporine as part of anti-rejection therapy. Studies indicate that 21%–35% of renal transplant patients are affected [7]. Various antibiotics have been used for treatment of gingival overgrowth in people, particularly those of the azalid family. Azithromycin administration at 500 mg per day for three consecutive days has shown particularly successful results in reduction of gingival overgrowth; this is the most commonly prescribed therapeutic regime in people [11]. Although the exact reason for clinical benefit is uncertain, beneficial response may be due in part to the ability of azithromycin to block cyclosporine-induced cell proliferation and collagen synthesis while activating matrix metalloproteinase-2 in gingival fibroblasts, thereby reducing hyperplastic lesions [15]. Down-regulation of transforming growth factors (TGF) may also contribute to decreased overgrowth [16]; several studies have documented increased levels of TGFβ110 and TGFβ211 in patients with cyclosporine-induced gingival overgrowth. Additionally, the antimicrobial effects and decreased inflammation secondary to concurrent infection likely plays a role in clinical improvement. The mechanism of development of cyclosporine-associated gingival overgrowth however has not been well documented in dogs.

Long-term administration of azithromycin did not cause any adverse effects in any of the dogs treated. In people, there has not been evidence to show that azithromycin inhibits the cytochrome P450 enzyme system [17], although a single case report has noted a potential interaction leading to elevated cyclosporine blood levels in a man administered intravenous azithromycin [18]. Evidence is unclear however whether azithromycin or another concurrently administered medication led to elevated cyclosporine blood levels in this patient. It is clear that other macrolide antibiotics (e.g., erythromycin, clarithromycin) can competitively bind the cytochrome P450 enzyme resulting in increased circulating serum cyclosporine concentration, however this does not appear to be the case with the azalid subclass (e.g., azithromycin). Never the less, given the possibility of interaction of these two medications (albeit rather unlikely), side effects of increased cyclosporine concentrations should be monitored in dogs receiving the medications concurrently.

Significant clinical improvement of gingival overgrowth was noted in all patients within the first four weeks of therapy, however complete clinical resolution of gingival overgrowth necessitated from 4 to 14 weeks of continued azithromycin treatment. All dogs however experienced complete clinical resolution of gingival overgrowth within the time frame noted. This is in contrast to what has recently been reported in the prospective study [14]. In the eight dogs that received oral azithromycin, only one had complete resolution gingival overgrowth during the four week treatment period. In addition, only two other dogs had >50% reduction in their global dental scores. In this current case series, azithromycin treatment was continued until complete clinical resolution of gingival overgrowth. Doses ranged from 6.6 mg/kg to 10.8 mg/kg orally once daily; the target dose was approximately 10 mg/kg. In most dogs, resolution of gingival overgrowth was a permanent effect without recurrence noted, even with continued administration of cyclosporine.

Findings of this case series should be taken in light of potential confounding factors. Notably, in some patients, the cyclosporine dose was decreased along with concurrent administration of azithromycin. Which intervention led to the greatest clinical benefit in regards to gingival overgrowth resolution is uncertain in these cases. In other cases however, there was a clear benefit of azithromycin administration for cyclosporine-induced gingival overgrowth. In four of six cases the cyclosporine dose was unchanged (Case 2 on relapse, 3, 4, and 5). Together, these current findings and those of the prospective study [14], offer a medical treatment option or adjunct therapy to surgery for gingival overgrowth in dogs receiving cyclosporine when the drug cannot be reduced/discontinued. Further evaluation of optimal duration of azithromycin therapy, as well as the formulation used (oral tablet, compounded capsule, toothpaste), is warranted.

## 4. Conclusions

This series highlights a potentially beneficial medical treatment option for gingival overgrowth even when cyclosporine dose reduction is not possible or elected, without the need for surgical resection of proliferative gingival tissue.
